# The sensory ecology of caterpillars

**DOI:** 10.1007/s00359-025-01778-x

**Published:** 2025-11-07

**Authors:** Sam J. England, Callum F. McLellan, Rochelle J. Meah, J. Benito Wainwright, Lauren Sumner-Rooney

**Affiliations:** 1 Department of Evolutionary Morphology, Museum für Naturkunde – Leibniz Institute for Evolution and Biodiversity Science, Invalidenstraße 43, 10115 Berlin, Germany; 2 School of Biological Sciences, University of Bristol, Bristol, UK; 3 Centre for Biological Diversity, School of Biology, University of St Andrews, Fife, UK

**Keywords:** Neuroethology, Lepidoptera, Larvae, Vision, Hearing, Vibration detection, Touch, Electroreception, Chemoreception, Sensory pollution

## Abstract

Caterpillars (larval Lepidoptera) are one of the most ecologically and evolutionarily significant taxa on Earth. As both feeders and food, they shape the dynamics of enumerate ecosystems on land. Key to this prominent role in nature is the sensory systems that inform, guide, and trigger their behaviour. Gaining an understanding of caterpillar sensory ecology therefore promises to reveal fundamental insights into the broader principles of ecology and evolution, conservation and management, within and beyond the Lepidoptera. To facilitate such an understanding, here we review the existing literature on the sensory physiology and ecology of all currently recognised sensory modalities in caterpillars, namely vision, hearing, vibration detection, touch, electroreception, chemoreception, hygroreception, thermoreception, and graviception. In each of these sensory modalities, we also explore the current evidence surrounding the threat of anthropogenic sensory pollution. Taken together, this review reveals the great depth and breadth of research into caterpillar sensory ecology, making clear the value of caterpillars to neuroethology, but also of neuroethology to caterpillars. However, many of the attributes that caterpillars bring to neuroethological research are yet to be taken advantage of. For example, there is currently a lack of comparative sensory system studies on caterpillars, utilising their ecological diversity and existing phylogenetic data. We also highlight many considerable knowledge gaps, most pertinently, the need to identify the sensors responsible for each sensory modality in caterpillars, and to characterise the potential effects of sensory pollution across all of these modalities.

## Introduction and the ecological and evolutionary context of caterpillars

Caterpillars, the larval stage of butterflies and moths (Lepidoptera), are an almost ubiquitous ecosystem constituent on land (and sometimes water). They participate in, and in many cases drive, countless ecological and evolutionary interactions and processes. Thus, to understand caterpillars is to understand a considerable portion of the dynamics of terrestrial life. Their remarkable life cycle and natural history have captivated scientists, artists, and the general public for millennia, with some of the earliest written accounts appearing in the works of Aristotle (384 − 322 BCE) (Peck 1942). In the modern day, and beyond basic science, caterpillars are notable as targets of conservation efforts for threatened species (Pyle 1995; New 1997, 2004; Longcore and Osborne 2015) and, conversely, management practices for species that are agricultural pests, invasive, or medically significant to humans, pets, and livestock (Stiling 2002; Diaz 2005; Tobin et al. 2012; Battisti et al. 2017; Day et al. 2017; Backe et al. 2021). One of the best ways to understand an animal is to understand how it perceives its environment, through a realisation of the sensory worlds that it inhabits (see von Uexküll’s “Umwelten” (Caves et al. 2019). Therefore, applying a sensory ecological approach to the study of caterpillars promises to better inform our scientific grasp of their fundamental evolution and ecology, as well as their conservation and management (Madliger 2012; Dominoni et al. 2020; Garvey et al. 2020; Elmer et al. 2021).

In the opposite direction, identifying the mechanisms, functional roles, and evolutionary principles of sensory modalities is greatly advanced by studying animal groups with diverse, well documented, ecologies and behaviours. Caterpillars are an ideal model in this regard. To date, over 166,000 species of Lepidoptera have been described (Beccaloni et al. 2024), making them currently the second most speciose taxonomic order on the planet. Each of these species exists as a caterpillar for part of its lifecycle, thus there are over 166,000 kinds of caterpillar in the world. Although most of this diversity is concentrated in the tropics, caterpillars have successfully colonised nearly all terrestrial ecosystems (Wardhaugh 2014; Wagner and Hoyt 2022). Such extensive biogeographical diversity offers a rich foundation for testing specific hypotheses on the ecological context by which caterpillars utilise their senses. Moreover, the convergent evolution of traits (e.g. habitat choice; (Kawahara and Rubinoff 2013)) in combination with a relatively well-resolved phylogeny (Bazinet et al. 2013, 2017; Kawahara and Breinholt 2014; Kawahara et al. 2019; Mayer et al. 2021; Rota et al. 2022), provides a powerful comparative framework for investigating the macroevolutionary dynamics underlying sensory adaptation, and the degree to which sensory shifts follow predictable patterns.

Unlike adult Lepidoptera, caterpillars are non-reproductive, allowing researchers to exclude sexual selection as an adaptive explanation for their behavioural, morphological, and most pertinently, sensory, traits. This presents a valuable simplification of the typically complex interactions between natural and sexual selection pressures. For example, many of the extravagant colours and morphologies that caterpillars have evolved could easily be mistaken for sexual signals if caterpillars were reproductive, but instead we can confidently attribute them to natural selection pressures like defence (Tullberg and Hunter 1996; Greeney et al. 2012; Robinson et al. 2023; McLellan et al. 2023a). The same is true for caterpillar sensory systems, which have evolved exclusively to improve survival under natural selection, whether that be by avoiding predation, finding suitable hostplants, communicating with conspecifics for cooperation, or orienting within their habitat.

Caterpillars play a dual ecological role as both major herbivores and a critical food source for predators, both of which inevitably exert strong selection pressures on the evolution of their sensory systems (e.g. Schoonhoven 1987; Castellanos and Barbosa 2006). In many biomes, they purportedly eat more leaves and transfer more energy from plants to other animals than all other herbivores combined (Janzen 1988; Wagner and Hoyt 2022). Hostplant use is often species-specific (Dyer et al. 2007), and while caterpillars often hatch directly onto suitable hostplants, they are also known to detect and respond to plant-associated chemical and visual cues (Saxena and Khattar 1977; Khattar and Saxena 1978; Rieske and Townsend 2005; Dyer et al. 2007; Rharrabe et al. 2014). However, not all caterpillars are exclusively herbivorous; cannibalism is also pervasive, particularly in gregarious species where close proximity increases the likelihood of encounters (Richardson et al. 2010). An incredibly small minority of caterpillar taxa have even shifted towards obligate carnivory, as predators, parasites, or scavengers (Montgomery 1982, 1983; Pierce 1995; Rubinoff and Haines 2005; Rubinoff et al. 2025). For example, most caterpillars within the butterfly family Lycaenidae have lifecycles intricately tied to ant colonies, often preying on host larvae and pupae within the brood chamber (Pierce 1995; Pierce et al. 2002). Such complicated multi-trophic interactions almost certainly require a suite of sensory systems to be effectively established and maintained.

As prey, the profound impact of predation on the behavioural ecology and evolution of caterpillars is evident, for example, in the myriad protective colouration strategies displayed, ranging from camouflage for concealment to aposematic signalling for deterring predators (Lichter-Marck et al. 2015; Robinson et al. 2023; McLellan et al. 2023b). Caterpillar predators include a wide variety of taxa, from invertebrates (such as wasps and true bugs) to vertebrates (such as rodents and birds), each relying on a distinct suite of behaviours and sensory cues to locate and capture prey (Stireman and Shaw 2022; Wagner and Hoyt 2022). This in turn could influence the evolution of caterpillar sensory traits. For example, in the senses of touch, hearing, and vibration detection, caterpillars exhibit sensitivity to different predator types based on differences in mechanosensory bending velocities or sound frequency respectively (Castellanos and Barbosa 2006; Castellanos et al. 2011; Breviglieri and Romero 2019). Resultingly, these senses can trigger predator-specific defensive behaviours (Castellanos and Barbosa 2006; Castellanos et al. 2011; Breviglieri and Romero 2019).

Shifts in diel activity and habitat between species further exposes caterpillars to distinct predator guilds as well as environmental sensory information, which may drive corresponding shifts in sensory investment and innovation (Seifert et al. 2016). A small number of species within the Cosmopterigidae, Crambidae, and Erebidae have even evolved associations with aquatic plants, giving them sometimes exclusively underwater lifestyles (Mey and Speidel 2008; Rubinoff and Schmitz 2010; Pabis 2018), which undoubtedly influences the sensory information available to these species, as well as the viable mechanisms required to detect it.

Lastly, the degree of sociality varies significantly amongst larval Lepidoptera, ranging from entirely solitary existences to highly gregarious lifestyles exhibiting communication systems and cooperative behaviours (Fitzgerald and Costa 1999; McLellan and Montgomery 2023; McLellan et al. 2023a). Such gregariousness has evolved convergently multiple times, with independent origins well in excess of 40 in the butterflies alone (Sillen-Tullberg 1988; Tullberg and Hunter 1996; McLellan and Montgomery 2023; McLellan et al. 2023a; Cicconardi et al. 2025). Unsurprisingly, these social behaviours share evolutionary trajectories with a multitude of other traits (Sillen-Tullberg 1988; Tullberg and Hunter 1996; McLellan and Montgomery 2023, 2024; McLellan et al. 2023a; Cicconardi et al. 2025), and sensory systems are undoubtedly amongst these too, given the divergence in sensory ecologies associated with group-living and sociality.

**Fig. 1:**
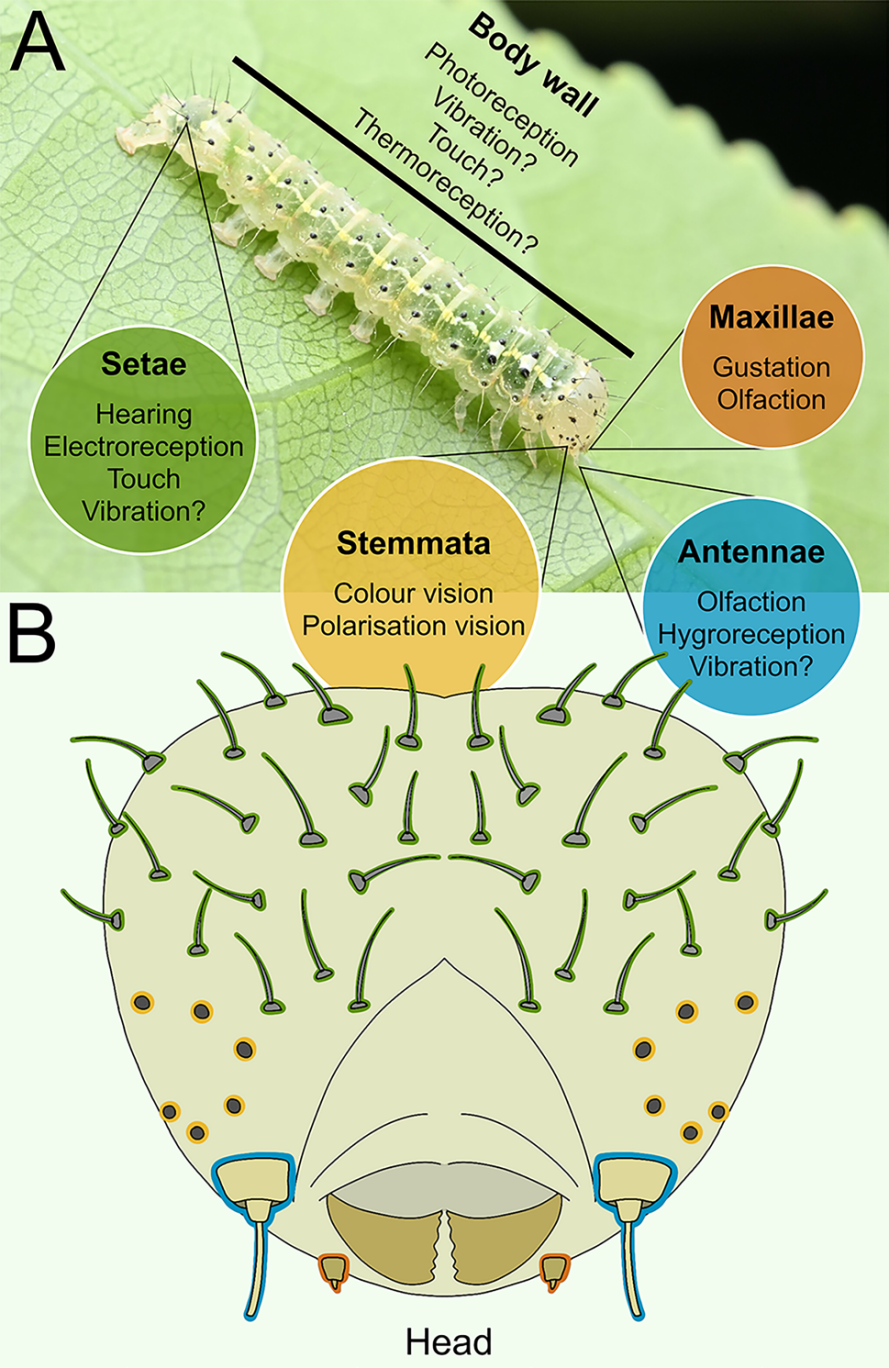
The sensory atlas of a caterpillar. Colours denote key sensory structures (dark green = mechanosensory setae, yellow = stemmata, blue = antennae, orange = maxillae). Text denotes sensory functions, “?” indicates that the sensory structure is a candidate for modality but is awaiting experimental confirmation as the mechanism. Labels are not exhaustive. **(A)** Labelled photograph of a Mahoe-stripper moth caterpillar (*Feredayia graminosa*, Noctuidae) in Pūponga, New Zealand, courtesy and copyright of Saryu Mae. **(B)** Illustration of the caterpillar head with key sensory structures highlighted, courtesy of Lucille Rose. Please note that not all setae are sensilla/possess a sensory function

All together, it is clear that the study of caterpillars stands to benefit greatly from the perspective of sensory ecology, and conversely, that sensory ecology stands to benefit greatly from the study of caterpillars. To facilitate this mutual benefit further, we have herein collated and synthesised the current knowledge of caterpillar sensory ecology, covering their array of senses (Fig. 1), including vision (ocular and extraocular, colour and polarization), hearing, vibration detection, touch, electroreception, chemoreception (olfaction and gustation), hygroreception, thermoreception, and graviception, as well as the potential impact of anthropogenic sensory pollution on these systems. In doing so, we identify common themes and distinctions amongst the senses and point to notable gaps in the current literature that warrant future exploration.

## The senses of caterpillars

### Vision and photoreception (light sensing)

Caterpillars have simple eyes, known as stemmata (Fig. 1). These eyes are sometimes referred to as ocelli, but this term is avoided here to prevent confusion with the ocelli of adult Lepidoptera, to which they are not thought to be homologous (Stehr 2009), or with spots of defensive colouration borne by many caterpillars, which superficially resemble eyes but have no capacity for vision (Janzen et al. 2010; Greeney et al. 2012; Hossie and Sherratt 2012, 2013; Skelhorn et al. 2014; Hossie et al. 2015).

The majority of caterpillars possess six bilateral pairs of stemmata (Gilbert 1994), but some early-diverging families (Kawahara et al. 2019) dissent from this (Gilbert 1994). For example, the Heterobathmiidae bear seven pairs (Kristensen and Nielsen 1983; Kristensen 1984), which may be the plesiomorphic state (Paulus and Schmidt 1978; Kristensen 1984), whilst the Micropterigidae sometimes have only five pairs (Tillyard 1923; Paulus and Schmidt 1978), the Eriocraniidae a single pair (Warrant et al. 2003), and the Agathiphagidae exhibit just the vestigial lensless remnants of two pairs (Kristensen 1984). Beyond these phylogenetic variances, it is also suggested that stemma loss is associated with leaf-mining behaviour in species of various families (Gilbert 1994).

**Fig. 2:**
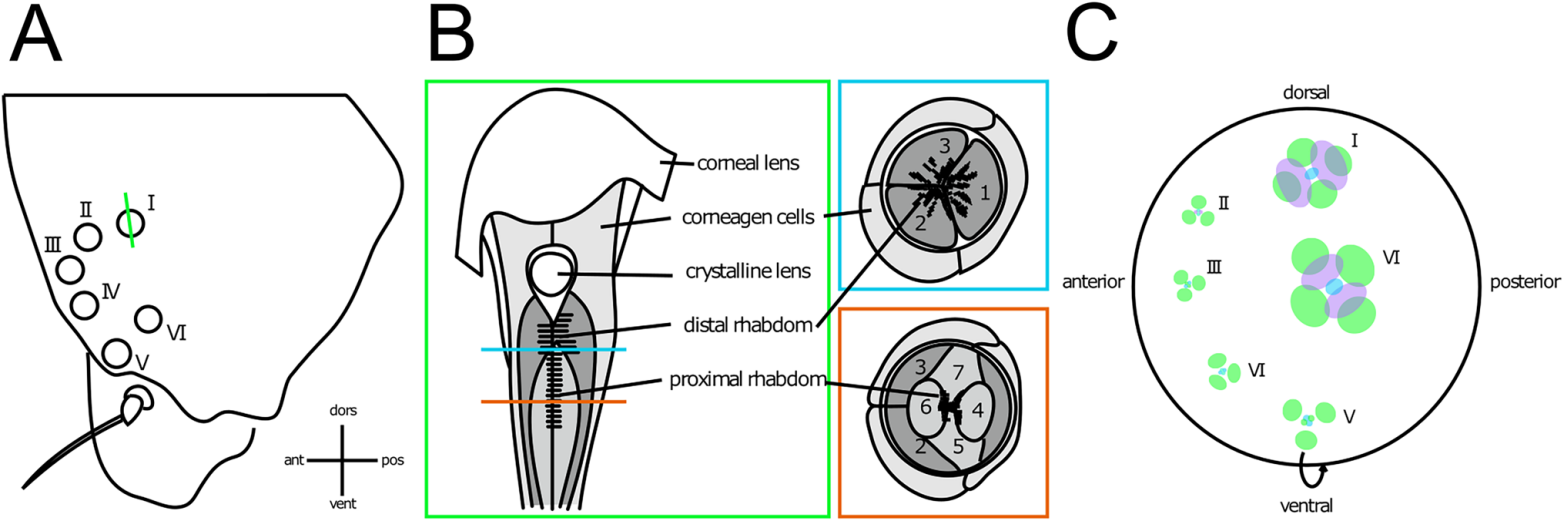
The visual system of caterpillars. Green, blue, and orange lines/boxes denote cut planes. **(A)** Typical arrangement and conventional numbering system of caterpillars with the usual six stemmata. **(B)** Example cross-sectional diagrams of the lens system and single rhabdom formed from the rhabdomeres of seven photoreceptors. These photoreceptors are arranged in two tiers and have different spectral sensitivities. **(C)** Receptive fields of each photoreceptor of each stemma; shown are green, blue, and UV-sensitive (in purple). Redrawn based on previous works (Ichikawa and Tateda 1982; Ichikawa 1991; Land and Chittka 2013).

The stemmata are seemingly relatively basic eyes, with each stemma approximately resembling the anatomy of a single ommatidium from the compound eyes of adult insects (Dethier 1942; Gilbert 1994; Lin et al. 2002). They are typically arranged in a loose C.-shape configuration on each side of the head, with one stemma positioned slightly further away (‘.’) from the rest that form the ‘C’ arrangement (Fig. 2A). Stemmata are dioptric, with an outer cuticular lens above a crystalline cone that is surrounded by three pigment cells (Dethier 1942, 1943; Gilbert 1994; Lin et al. 2002) (Fig. 2B). Incident light then reaches the rhabdom, which consists of seven photoreceptors arranged in a tiered structure, with either three proximal and four distal or vice versa (Li and Chang 1991; Gilbert 1994; Lin et al. 2002) (Fig. 2B). The configuration of these layers (3–4 vs. 4−3) varies both between species, and between stemmata of the same species (Li and Chang 1991; Gilbert 1994; Lin et al. 2002). The photoreceptor cells contain pigment granules that migrate away from the central rhabdom in dark-adapted stemmata (Gilbert 1994).

The efferent axons from each photoreceptor and each stemma form a single optic nerve bundle, which projects to the larval brain. The larval optic lobes include a lamina and medulla, and are reduced and superseded by the adult optic neuropils during metamorphosis (Huetteroth 2010). Each stemma has a dedicated cartridge in the lamina, and their relative positions broadly preserve their spatial arrangement on the head capsule (Ichikawa and Tateda 1984). In *Papilio xuthus* (Papilionidae), the distal, green-sensitive axons terminate in the lamina, and the proximal axons terminate in the medulla, where signals from within and between stemmata are combined via both spatial antagonism and colour opponency (Ichikawa 1986).

The distal photoreceptors within each stemma have larger and more divergent receptive fields (around 6.5° from the overall axis of the stemma in both *Papilio xuthus*, Papilionidae (Ichikawa and Tateda 1982) and *Pieris brassicae*, Pieridae (Barrer 1969) than the proximal photoreceptors, whose receptive fields often overlap and may coincide with the overall axis of the stemma (Barrer 1969; Ichikawa and Tateda 1982). However, as each stemma possesses a single rhabdom, they are not thought to be individually capable of spatial resolution and are functionally analogous to single ommatidia (Dethier 1943).

Instead, caterpillars are thought to attain spatial information from between stemmata. The fields of view of adjacent stemmata do not overlap (Fig. 1C), theoretically producing a very coarse, disjointed mosaic view of intensities from different directions (Dethier 1943). Accordingly, Dethier reported that ‘form vision’ (see below) was significantly impaired in animals with all but one stemma occluded, but phototaxis was not. This presumably precludes complex visual behaviours, although it has been suggested that the stereotypical head movements of caterpillars may allow them to sample a larger visual field and generate crude form vision (Dethier 1943; Gilbert 1994), and caterpillars do perform these scanning head movements more frequently when an object is within their visual field (Götz 1936).

Trichromatic sensitivity (green, blue, UV) has been demonstrated in the stemmatal system of *Mamestra brassicae* (Noctuidae), *Pieris rapae* (Pieridae), *Papilio xuthus* (Papilionidae), and *Bombyx mori* (Bombycidae) (Ichikawa and Tateda 1982). Different stemmata express different combinations of opsins (Fig. 2C); the distal photoreceptors generally being responsive to green light, and the proximal ones to a combination of blue, green, and UV (Ichikawa and Tateda 1982). Where there are four proximal photoreceptors, they are often paired in their spectral sensitivity (Ichikawa and Tateda 1984). The swallowtail butterfly caterpillar (*Papilio*) has been well studied in the context of this trichromacy (Gilbert 1994), and it appears that the processing of colour information is quite sophisticated. Neural opponency in the medulla acts both between wavelength-sensitive classes of neurons within stemmata, and within wavelength-sensitive classes of neurons between stemmata (Ichikawa 1986).

The flicker fusion frequency of caterpillar stemmata has been identified as around 25–30 Hz in *Bombyx mori* (Bombycidae) and *Pieris* sp. (Pieridae) (Ishikawa and Hirao 1960; Gilbert 1994), which is comparatively slow for insects (Ruck 1958). While this would limit the value of stemmata in supporting fast visual behaviours such as predator detection, it is presumably sufficient for orientation to stationary stimuli and detecting polarized light.

The ecological and behavioural roles of vision in caterpillars remain to be fully explored, but positive and negative phototaxes have been demonstrated in countless species. These behaviours may facilitate feeding, moulting, and pupation site selection (de Ruiter and van der Horn 1957a; Inoko et al. 1981; Shields and Wyman 1984; Taylor and Shields 1989; Royer et al. 2021), and help avoid cannibalism (Taylor and Shields 1989). They could also inform dispersal behaviours, allowing younger caterpillars to more easily balloon (Taylor and Shields 1989), as well as direct the orientation of travelling processionary caterpillars, which navigate preferentially to dark or light regions of their field of view depending upon the species (Uemura et al. 2020). The direction and extent of phototaxis in caterpillars is also context-dependent, and appears to vary with developmental stage, generally transitioning from photopositivity to photonegativity through increasing instars, supporting the ecological relevance of these behaviours, in that young caterpillars may wish to climb towards light for food, whilst later instars could be driven towards darkness to find suitable pupation sites (de Ruiter 1956; de Ruiter and van der Horn 1957a; Ruiter and Horn 1957b; Beetsma et al. 1962; Madge 1964a, b; Olson and Rings 1969; Archer and Musick 1976; Shields and Wyman 1984; Mariath 1984; Taylor and Shields 1989; Royer et al. 2021). Environmental conditions like temperature and food availability also influence caterpillar phototaxis (Wellington 1960; Madge 1964a, b; Shimizu et al. 1976; Shimizu and Kato 1978; Kavaliers and MacVean 1980; Inoko et al. 1981; Shields and Wyman 1984; Mariath 1984; Gilbert 1994), and pathogenic infection very likely plays a role (van Houte et al. 2014; Houte et al. 2015; Han et al. 2018; Bhattarai et al. 2018a, b; Gasque et al. 2019), though this last claim has been disputed (Dobson et al. 2015). Phototaxis is apparently still performed unhindered by caterpillars with all but one stemma occluded (Oehmig 1940; Dethier 1943). Interestingly, phototaxis in *B. mori* caterpillars (Bombycidae) can be suppressed by the olfactory system, for example when detecting chemical cues from foodplants (Shimizu and Kato 1978; Inoko et al. 1981). However *P. demoleus* caterpillars (Papilionidae) prioritise attractive visual stimuli over attractive olfactory stimuli (Khattar and Saxena 1978), suggesting that the principles of sensory integration in caterpillars are complicated and species-specific. Further, attraction to certain colours in caterpillars has been demonstrated and associated with the selection of pupation sites (Starnecker 1996).

Beyond simple phototaxis, experiments in *Papilio demoleus* (Papilionidae) caterpillars show a visual attraction to sheets of *Citrus* leaves, as well as foliage green coloured paper sheets, but this attraction depends only on the visual angle subtended by the sheets, and not on the actual dimensions of the sheets (Saxena and Khattar 1977; Khattar and Saxena 1978), suggesting rather simple visual processing. Nonetheless, caterpillars seem able to use contrast, reflectance, or shape cues to orient towards and select feeding sites, being attracted to vertical black objects presumably reminiscent of plant stems or tree trunks (Rieske and Townsend 2005). These behavioural reports also include choice experiments between various dark shapes against a light background (Hundertmark 1937; Rieske and Townsend 2005). Hundertmark found that caterpillars preferentially moved towards dark shapes that were larger overall, and larger at the bottom than the top (Hundertmark 1937). Although these findings could be ascribed to phototaxis, and not form vision, he also reported that caterpillars preferred some aspect ratios to others, and vertical edges to diagonal or horizontal ones. Additional experiments are necessary to shed more light on whether the stemmata mediate visual (as opposed to simply phototactic) behaviour. Unfortunately, caterpillars appear to not exhibit an optomotor response (Schlegtendal 1934), making behavioural assays of their visual capabilities challenging.

Stemmata also endow some caterpillar species with polarization vision (Wellington et al. 1951; Wellington 1955; Doane and Leonard 1975; Dethier 1989; Li and Chang 1991; Uemura et al. 2021a). Evidence of polarization vision, either anatomically or behaviourally, has been documented in caterpillars of the families Notodontidae (Uemura et al. 2021a), Noctuidae (Li and Chang 1991), Erebidae (Doane and Leonard 1975; Singleton-Smith 1980; Dethier 1989), Lasiocampidae (Wellington et al. 1951; Dethier 1989), Tortricidae (Wellington et al. 1951; Wellington 1955), Geometridae (Wellington 1955), Lasiocampidae (Wellington 1955), Nymphalidae (Wellington 1955), and Papilionidae (Wellington 1955; Toh and Sagara 1982), but it has only been empirically demonstrated in the Notodontidae (Uemura et al. 2021a), Erebidae (Doane and Leonard 1975), Lasiocampidae (Wellington et al. 1951), and Tortricidae (Wellington et al. 1951; Wellington 1955).

The polarization of light describes the direction and distribution of oscillation of the electric field vectors (e-vector) of the electromagnetic waves within a beam of light. An eye will only detect polarized light if its light-sensitive visual pigments are orientated parallel to the angle of e-vector oscillation. As such, the angular geometry and arrangement of these visual pigments within an eye is an inherent characteristic for the detection of the polarization of light. In stemmata, the visual pigments are found in tube-like microvillar projections from the photoreceptor cells in the rhabdom. In the 3 or 4 proximal photoreceptor cells of stemmata I (Li and Chang 1991; Uemura et al. 2021a), VI (Li and Chang 1991), and possibly III (Singleton-Smith 1980), the geometry of these microvilli naturally aligns the visual pigments in a single orientation and thus creates ‘built-in’ dichroism, i.e., an ability to selectively absorb polarized light of different e-vector angles. The level of polarization sensitivity of lepidopteran caterpillars is currently unknown but the ecologically similar larva of the sawfly, *Perga*, has a relatively high average polarization sensitivity of 6 (Meyer-Rochow 1974; Labhart 1980, 1986; Dacke et al. 2002).

Caterpillars use their polarization vision for detection of the skylight polarization pattern, a ubiquitous compass cue used by many insects (Krapp 2007), when dispersing in search of food or suitable pupation sites (Wellington et al. 1951; Wellington 1955; Uemura et al. 2021a). The skylight polarization pattern is a pattern of polarized light in the sky created by Rayleigh scattering of sunlight or moonlight in the Earth’s atmosphere. The combined e-vector axes of the scattered light create a reliable and distinctive pattern of polarized light in the sky that moves relative to the diel movements of the sun and the moon. Polarization-sensitive animals use this pattern for orientation either as a wide-field visual landmark or as a true celestial compass cue and can do so even if either celestial body is occluded from view. Indeed, dispersing caterpillars will switch between using the polarization pattern and the solar azimuth as the dominant orientation cue depending on the relative reliability of either (Wellington et al. 1951; Wellington 1955; Doane and Leonard 1975), or if heat stressed and thus shade-seeking (Wellington et al. 1951). This is an advantageous behavioural mechanism in navigation as celestial cues are dynamic and change with the diel movement of celestial bodies, cloud cover, or become obstructed from view as individuals move around their environment. In processionary caterpillars of the family Notodontidae, mature larvae travel to pupation sites in a single file, but only the lead caterpillar orientates using the polarization pattern, the remainder of the procession using physical contact and pheromone cues to stay in file (Fitzgerald 2003; Steinbauer 2009; Uemura et al. 2021a).

The polarization-sensitive stemmata in some species have specialized dioptric structures on the skyward-facing part of the lens, such as y-shaped grooves (Singleton-Smith 1980; Li and Chang 1991) or a rugged surface (Singleton-Smith 1980; Uemura et al. 2021a), thought to facilitate and stabilise the detection of the skylight polarization pattern by enlarging visual fields and decreasing acuity (i.e. reducing visual clutter) (Singleton-Smith 1980; Uemura et al. 2021a). As well as physiological adaptations, dispersing caterpillars will use behaviour to facilitate the detection of the skylight polarization pattern for orientation. Caterpillars will perform scan-like head movements following a change in overhead polarization and will correct their heading directions accordingly (Wellington et al. 1951; Dethier 1989), a common behaviour in polarization-guided insect navigation (Rossel and Wehner 1984; Baird et al. 2012; Grob et al. 2022).

In addition to vision with their eyes, it has been shown that multiple species of caterpillar are afforded extraocular photoreception (Poulton 1890; Kato et al. 1989; Eacock et al. 2019). Via stemmata occlusion experiments, it was demonstrated that the detection of light spectra through dermal photoreceptors facilitates colour change in caterpillars, allowing them to better match their backgrounds, improving camouflage (Eacock et al. 2019). Further, earlier experiments indicated the role of extraocular photoreception at the caterpillar stages in determining subsequent pupal colour (Poulton 1890; Kato et al. 1989), again for the sake of increasing survival through crypsis (Wiklund 1975; Hazel et al. 1998). It is likely that this mechanism explains many of the light-dependent colour polymorphisms seen in a wide variety of lepidopteran larvae and pupae (Poulton 1887; Wiklund 1972; Smith 1980; Smith et al. 1988; Grayson and Edmunds 1989; Mayekar and Kodandaramaiah 2017). Opsin expression within the caterpillar dermis indicates trichromatic sensitivity (likely UV, blue, and green), although the exact machinery utilised for extraocular vision awaits identification (Eacock et al. 2019). The stemmata also seemingly contribute to the detection of environmental spectra to inform colour change but are likely secondary to the dermal photoreceptors in this task (Eacock et al. 2019). Intriguingly, caterpillars with all of their stemmata occluded can still crudely orient based on light, suggesting that the extraocular light sense may also contribute to phototaxis (Oehmig 1940).

### Hearing (auditory sensing)

Hearing has been reported in many species of caterpillar across numerous families, including the Erebidae (Minnich 1936), Geometridae (Minnich 1936), Lasiocampidae (Myers and Smith 1978), Noctuidae (Minnich 1936; Markl and Tautz 1975; Tautz 1977, 1978; Tautz and Markl 1978; Tautz and Rostás 2008), Notodontidae (Abbott 1927; White et al. 1983), Nymphalidae (Minnich 1925, 1936; Rothschild and Bergström 1997; Davis et al. 2018; Taylor and Yack 2019; Lee et al. 2021), Papilionidae (Minnich 1936), Pieridae (Minnich 1936), and Saturniidae (Minnich 1936; Breviglieri and Romero 2019), with many more likely to be discovered (Minnich 1936; Yack 2022). All of the species identified as sensitive to sound react behaviourally to low-frequency airborne sound, approximately in the range of 40–1000 Hz (Markl and Tautz 1975; Taylor and Yack 2019; Yack 2022), but in some cases up to 2100 Hz (Myers and Smith 1978). Behavioural responses to these sounds include thrashing, flicking, freezing, dropping, ultrasound production, and volatile emission (Yack 2022), which are all known defensive strategies employed by caterpillars (Greeney et al. 2012). The strongest reactions are generally observed in response to frequencies of around 100–200 Hz (Markl and Tautz 1975; Tautz 1977; Taylor and Yack 2019), which corresponds to the typical wingbeat frequencies of insects (Sotavalta 1963; Byrne et al. 1988; Rashed et al. 2009; Deakin 2010; Ha et al. 2013), most notably the wasps that so incessantly attempt to predate and parasitise caterpillars (Sotavalta 1963; Tautz and Markl 1978; Rashed et al. 2009; Stireman and Shaw 2022; Wagner and Hoyt 2022). Together these facts strongly insinuate that the primary function of hearing in caterpillars is to detect their predator and parasitoid natural enemies. Indeed, caterpillars with their sound-detecting sensilla ablated were predated upon by wasps significantly more than those with intact sensilla in an experimental microcosm (Tautz and Markl 1978). Given that defensive reaction rates vary between responses to audio playbacks of predatory wasps and birds, as well as mosquitos that are presumably ecologically unimportant to caterpillars (Breviglieri and Romero 2019), it may even be the case that caterpillars can determine the identity of airborne sound sources via auditory spectral information.

**Fig. 3:**
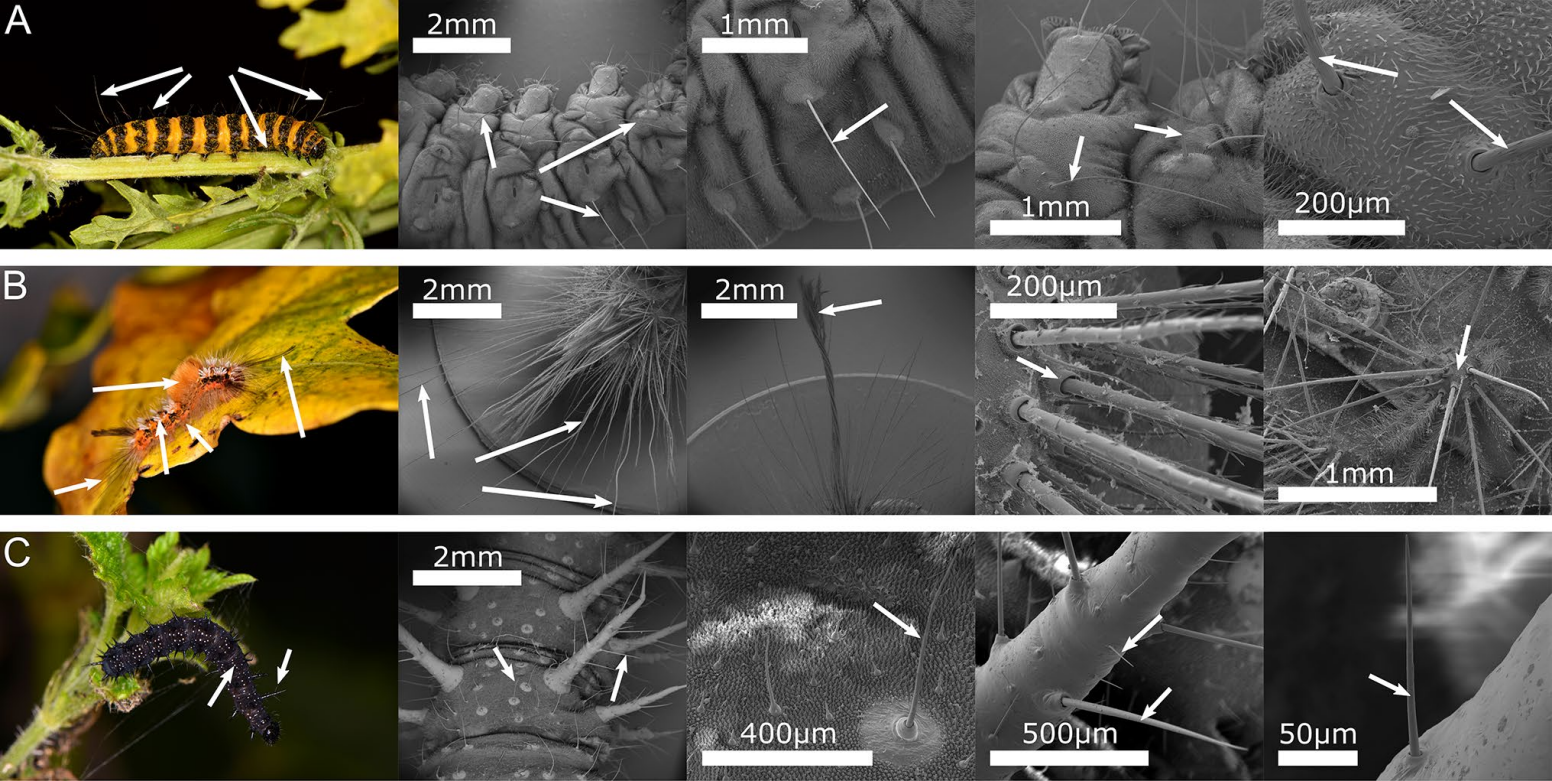
Examples of setae found on caterpillars, imaged with macrophotography and scanning electron microscopy. Many are mechanosensory (observe articulation at their base) and thus implicated in hearing, touch, electroreception, and possibly vibration detection. Arrows point to example setae. Rows contain single species: **(A)** Cinnabar moth caterpillar, *Tyria jacobaeae*, Erebidae. **(B)** Scarce vapourer moth caterpillar, *Telochurus recens*, Erebidae. **(C)** European peacock butterfly caterpillar, *Aglais io*, Nymphalidae. Modified from previous work (England and Robert 2024a). Please note that not all setae are sensilla/possess a sensory function

The sensilla responsible for hearing are not known for the majority of caterpillars, but in the exceptions to this, mechanosensory trichoid (hair-like) setae have been implicated (Markl and Tautz 1975; Taylor and Yack 2019). Through ablation experiments, the hearing organs in cabbage moth caterpillars (*Mamestra brassicae*, Noctuidae) have been experimentally identified as eight thoracic trichoid sensilla, and in monarch butterfly caterpillars (*Danaus plexippus*, Nymphalidae) just two (Taylor and Yack 2019), though other mechanosensory setae may contribute in an auxiliary fashion (Taylor and Yack 2019). These sensilla are approximately 0.5 mm long, and innervated at the base such that sound reception is achieved when they are mechanically deflected by the near-field particle velocity component of airborne sounds (Markl and Tautz 1975; Tautz 1977, 1978; Tautz and Markl 1978; Taylor and Yack 2019; Yack 2022). In insects, setae sensitive to mechanical deflection, and thus potentially sound, can be identified by their protrusion from sockets at their base on the body cuticle (Keil 1997). Such sockets generally possess an elastic joint membrane that facilitates articulation of the seta (Keil 1997). These features distinguish mechanosensory setae from unarticulated setae that function as defensive physical barriers or chemoreceptors in some species (Sugiura and Yamazaki 2014; Wagner and Hoyt 2022). Remarkably, these sound-detecting sensilla remain sensitive during the moulting stage between caterpillar instars, except for the 30–60 min that actual ecdysis takes place (Gnatzy and Tautz 1977). Tympanal ears are not known in any caterpillar species (Yack 2022) and thus lepidopteran larvae are probably unable to detect the far-field, pressure component of sounds. Only one sensillum is required for the caterpillars to detect airborne sound, but the animal’s sensitivity is reduced as increasing numbers of sensilla are removed (Markl and Tautz 1975). These sensilla are rather sensitive, and are reportedly capable of detecting a wasp wingbeat from up to 70 cm away (Tautz 1978; Tautz and Markl 1978). Given the almost ubiquity of mechanosensory hairs among caterpillars (Fig. 3), it seems likely that these are responsible for hearing in a great number of species. The same sensilla appear to also be involved in electroreception (see ‘Electroreception’ below) (England and Robert 2024a) and the two senses may constructively interfere to boost caterpillars’ sensitivity to the wingbeats of their natural enemies.

Hearing has not been investigated in any of the aquatic caterpillar lineages, but if present, would likely rely upon different mechanisms due to the differing fluid mechanics of water compared to air. Additionally, the predatory *Eupithecia* (Geometridae) caterpillars of Hawaiʻi are capable of ambushing flighted insects (Montgomery 1982, 1983; Sugiura 2010), and would thus presumably find great utility in detecting the wingbeats of their prey. However, they purportedly only strike when specialised elongate setae are triggered by tactile stimuli (Montgomery 1982, 1983). Nonetheless, future studies should aim to probe for sound detection in this predatory clade, in the pursuit of broadening the ecological functions known for hearing in caterpillars.

Lastly, many caterpillars produce ultrasound in response to approaches or attacks from predators (Yack 2022), and it has been suggested in gregarious caterpillar species that these high frequency noises could serve as communication signals to conspecifics, in order to warn others and coordinate collective defence strategies (Breviglieri and Romero 2019; Yack 2022). However, no such mechanism for ultrasonic hearing has been identified in caterpillars thus far, and it is unlikely that the trichoid sensilla utilised for low-frequency hearing would be suitable for this task.

### Vibroreception (vibration sensing)

The detection of vibrations, typically defined in a biological context as acoustic cues and signals transmitted through a solid substrate like a plant or the ground, is also thought to be widespread amongst caterpillars (Yack et al. 2001; Yack 2022; Yack and Yadav 2022). Vibration sensitivity has been attributed so far to the Drepanidae (Yack et al. 2001, 2014; Bowen et al. 2008; Scott et al. 2010; Guedes et al. 2012; Scott and Yack 2012; Yadav et al. 2017; Yadav and Yack 2018; Matheson et al. 2025), Gelechiidae (Sigmon 2015; Yack and Yadav 2022), Geometridae (Castellanos and Barbosa 2006), Gracillariidae (Meyhöfer et al. 1997; Djemai et al. 2001; Fletcher et al. 2006), Noctuidae (Turchen et al. 2023), Oecophoridae (Hunter 1987; Sigmon 2015; Yack and Yadav 2022), and Tortricidae (Yack and Yadav 2022), though many other families are likely capable too.

Given that caterpillars are flightless and thus obligately bound to the substrate, the potential value of vibration sensing in these larvae is immediately obvious. In comparison to the hearing of airborne sounds, which is thus far known only to be used for detecting natural enemies, the functional breadth of vibration sensing in caterpillars is slightly greater (Yack 2022; Yack and Yadav 2022). This is especially true given the ability of caterpillars to produce substrate-borne vibrations themselves, and thus vibration sensing can contribute to communication, not only passive sensing of environmental cues (Scott et al. 2010; Yack 2022; Yack and Yadav 2022). Studies so far have identified a role for vibration sensing by caterpillars in territorial interactions (Hunter 1987; Yack et al. 2001, 2014; Fletcher et al. 2006; Bowen et al. 2008; Scott et al. 2010; Guedes et al. 2012; Scott and Yack 2012; Sigmon 2015; Matheson et al. 2025), predator/parasitoid detection (Meyhöfer et al. 1997; Djemai et al. 2001; Castellanos and Barbosa 2006; Guedes et al. 2012), and recruitment by conspecifics to feeding and shelter sites (Yadav et al. 2017; Yadav and Yack 2018). The number of plausible functions is much greater, however, with suggestions including receiving vibrational cues and signals from mutualist ants, echolocation, and conspecific social signals such as indicating intent, coordinating feeding turns, and establishing leadership (Yack and Yadav 2022).

Despite the clear behavioural role of vibration sensing in caterpillars, the sensory mechanism behind this ability is not yet known (Yack 2022; Yack and Yadav 2022). Based on anatomical surveys of caterpillar bodies, informed by knowledge of vibration detectors in adult insects and other taxa, it has been suggested that mechanosensory setae (Fig. 3) and/or chordotonal organs are implicated (Yack and Yadav 2022), but this awaits neurophysiological and behavioural confirmation.

Regardless, it is known that vibration detection by caterpillars is likely quite sensitive and somewhat sophisticated. Caterpillars can detect vibrational stimuli as small as that generated by the insertion of a parasitoid’s ovipositor at some distance away (Meyhöfer et al. 1997; Djemai et al. 2001), and some species have been shown to be capable of distinguishing the vibrational cues of different predator types, other herbivores, and abiotic sources like rain and wind (Castellanos and Barbosa 2006; Guedes et al. 2012; Turchen et al. 2023).

### Touch and fluid flow detection (tactile and flow sensing)

Despite being one of the most widespread senses in the animal kingdom, touch, the mechanical detection of direct contact, has received surprisingly little attention in studies on caterpillar sensory ecology. Experiments and observations suggest that caterpillars respond behaviourally to contact-bending of mechanosensory setae (Fig. 3) (Frings 1945; Montgomery 1982, 1983; Castellanos et al. 2011), and also touch and pressure on the body wall (Frings 1945). In most caterpillar species tested, the reactions to touch consist of defensive flailing or flicking (Frings 1945), dropping or fleeing (Castellanos et al. 2011), coiling (England and Robert 2024a), or defensive sound production (Low et al. 2023, 2024), suggesting touch is primarily utilised in anti-predator functions. Indeed, the mechanosensory setae of *Orgyia leucostigma* (Erebidae) elicit specific behavioural responses to touch stimuli from different predator types (Castellanos et al. 2011). These distinguishments between predator types appear to be possible via touch due to differences in the bending velocities of the setae caused by different predators (Castellanos et al. 2011). As with the sensilla responsible for hearing in caterpillars, the mechanosensory setae that contribute to their sense of touch can be identified morphologically by the presence of an articulated socket in the body wall (Keil 1997).

Additionally, the predatory *Eupithecia* (Geometridae) caterpillars of Hawaiʻi reportedly trigger their ambushing strikes when prey make contact with their mechanosensory setae (Montgomery 1982, 1983), pointing to a co-option of this originally anti-predator sensory system into a predatory one. It is likely that specific adaptations have arisen in the Hawaiian *Eupithecia* mechanosensory system to increase their sensitivity to touch stimuli from prey.

Outside of predator-prey interactions, there is strong evidence that the ability of processionary caterpillars to maintain their single-file processions is partly mediated by positive thigmotaxis from the setae at the posterior tip of the caterpillar in front touching the caterpillar behind (Fitzgerald 2003; Steinbauer 2009). Caterpillars of *Thaumetopoea pityocampa* (Notodontidae) can be very reliably recruited to follow by touching them with the dismembered posterior caterpillar sections bearing setae, but not a stick or paintbrush, suggesting this tactile conspecific recognition may be somewhat sophisticated (Fitzgerald 2003). Additionally, the re-introduction of *Ochrogaster lunifer* (Notodontidae) caterpillars with their posterior setae cut, back into the procession, results in a breakage of the procession (Steinbauer 2009). This is apparently due to the lack of tactile stimuli available to the following caterpillar, now that there are no setae to make contact with, as well as the caterpillar in front being unable to detect the breakage behind them now that its setae are cut, resulting in it not stopping when detachments occur (Steinbauer 2009).

In the cases of touch sensitivity directly on the body wall, the receptors involved have not been identified. They may simply be the smaller setae generally distributed throughout the caterpillar dermis (Fig. 3), not usually visible to the naked eye, or some other structure that could act as a pressure sensor, such as campaniform sensilla, the subepidermal nerve-net, or proprioceptors (Frings 1945). Disentangling these responses from the sensation of noxious stimuli (nociception), which elicit similar defensive behaviours (Frings 1945; Walters et al. 2001; van Griethuijsen et al. 2013; Caron et al. 2020), presents a further challenge. Interestingly, *Manduca sexta* (Sphingidae) caterpillars utilise the same neurons for detection of both noxious mechanical stimuli and noxious thermal stimuli, pointing to multimodality and/or co-option (Caron et al. 2020), but such mechanisms are likely different from those for non-noxious stimuli. Future work should aim to validate the early experiments on dermal touch sensitivity and interrogate its potential mechanisms further.

In addition to detection of direct tactile stimuli, it is also likely that caterpillars can perceive fluid flow across their bodies. Caterpillars of numerous species are avid ballooners, using silk strands as aerial sails to disperse (Bell et al. 2005). Given the apparent importance of wind and airflow to the success of this ballooning behaviour in caterpillars (Bell et al. 2005; Li et al. 2023) and other ballooning arthropods (Humphrey 1987; Weyman 1993; Bell et al. 2005; Reynolds et al. 2007; Cho 2021; Narimanov et al. 2021; Montes and Gleiser 2025), it seems likely that caterpillars may be able detect such fluid flow, most likely via their mechanosensory setae, and utilise it as a sensory cue to make dispersal decisions and trigger the behaviour. However, the sensory ecology of this process is poorly explored in caterpillars and awaits experimental characterisation.

### Electroreception (electrostatic sensing)

The sense most recently discovered in caterpillars is electroreception (England and Robert 2024a): the detection of ecologically relevant electric fields (England and Robert 2022). This sense is sometimes more specifically referred to as ‘aerial electroreception’ when used in air (Clarke et al. 2017; England and Robert 2022; Robert 2024; England et al. 2025), as is the case for caterpillars (England and Robert 2024a). Caterpillars can detect the electric fields that emanate from their predators, such as wasps, and can use this sense to initiate or extend defensive behaviours such as coiling, freezing, flailing, or biting (England and Robert 2024a). Their predators emit these electric fields because many animals in the terrestrial environment naturally accumulate static charge, most likely through friction with the air, plants, or substrate, which then generates an electrostatic field around that animal (Clarke et al. 2013; Badger et al. 2015; Hunting et al. 2022b; England and Robert 2022, 2024b; England et al. 2023, 2025). Predators such as wasps also accumulate electrostatic charge (England and Robert 2024a; England et al. 2025), and thus have the potential to be detected by electrical means.

Electroreception has thus far been behaviourally confirmed in three species of caterpillar, *Aglais io* (Nymphalidae), *Tyria jacobaeae* (Erebidae), and *Telochurus recens* (Erebidae), but it is likely to be widespread amongst the Lepidoptera, as with hearing. Laser Doppler vibrometry performed on *Ty. jacobaeae* and *Te. recens* points to mechanosensory setae being the site of electroreception, with electrostatic deflections of these setae occurring at ecologically relevant electric field strengths and frequencies (England and Robert 2024a), though electrophysiological confirmation of this is required. The setae of *Ty. jacobaeae* exhibit an electromechanical resonance around the wingbeat frequency of most aerial insects, suggesting that they could be tuned to the wingbeats of their predators (England and Robert 2024a). Again, as with their senses of hearing and touch, candidate electroreceptors on caterpillars can be most easily identified by searching for mechanosensory setae, distinguished by their articulation via sockets at their base (Keil 1997). Whilst caterpillars do sometimes carry a small amount of electrostatic charge themselves (England and Robert 2024a), this is not a requirement for them to be able to detect the electric field of other animals, because the setae are deflected predominantly through being polarized by the source electric field (England and Robert 2024a). This is indicated by the setae maximally responding at double the electrical stimulus frequency (England and Robert 2024a), behaving in a phasic manner. Ecologically, this is important, because it suggests that caterpillars can be electrically ‘invisible’ or ‘camouflaged’ whilst still detecting electricity themselves.

As a very newly discovered sensory modality in any animal, let alone caterpillars, much more is still to be learned about the ecology and evolution of aerial electroreception. Outside of predator detection, it is possible that electroreception could be involved in other ecological contexts of caterpillars. For example, it could feasibly contribute to prey detection in the predatory species of caterpillars, or conspecific recognition in aggregating and processionary clades. Perhaps most promisingly, it has been demonstrated that the atmospheric potential gradient can provide additional electrostatic lift for ballooning spiders, and that the precursory behaviours to ballooning can be triggered in these spiders via sensory detection of the local electric field strength (Morley and Robert 2018; Narimanov et al. 2021). Given that many species of caterpillar also balloon (Bell et al. 2005), and that caterpillar electroreception to electric field strengths congruent with the typical atmospheric potential gradient has already been confirmed (England and Robert 2024a), it seems highly plausible that ballooning caterpillars may use their electrostatic sense in a similar way to ballooning spiders.

### Magnetoreception (magnetic sensing)

Magnetoreception, the detection of magnetic fields, has thus far not been demonstrated in any caterpillar species. This is perhaps to be expected, because magnetoreception is typically utilised for long distance navigation and orientation; tasks that the vast majority of caterpillars do not undertake. Nonetheless, the processionary caterpillars offer one possible ecological context in which magnetoreception could prove useful, in that detecting the Earth’s magnetic field could help these caterpillars to maintain their desired straight trajectories. Furthermore, since many adult Lepidoptera appear to have a magnetic sense (Robin Baker and Mather 1982; Robin Baker 1987; Srygley et al. 2006; Guerra et al. 2014; Xu et al. 2017; Dreyer et al. 2018, 2025), the genetic framework exists within the caterpillar genome to potentially allow them to build magnetoreceptors and detect magnetic cues too.

### Chemoreception (chemical sensing)

Caterpillar chemoreception plays a vital role in their ecology, and accordingly is one of the most extensively studied of their senses. Of the two main modes of larval chemoreception, olfaction (‘smell’) is primarily used for host plant location, whereas gustation (‘taste’), also referred to as contact chemoreception (Chapman 2003), is primarily used for host selection (Agnihotri et al. 2016). Both are important, yet gustation is better understood, having received comparatively more attention in the literature than olfaction (Wang et al. 2024). Larval chemoreception operates almost exclusively through structures on the head known as the maxillae (and their associated palpi) and the antennae (Schoonhoven and Van Loon 2002; Wang et al. 2024). Olfaction primarily occurs through the antennae, although a small number of olfactory receptors also exist on the maxillae (Hansson 1995; Wang et al. 2024), whereas contact chemoreception primarily occurs through the maxillae (Glendinning et al. 1998; Schoonhoven and Van Loon 2002). Each maxillary palpus holds eight chemosensory structures, three of which are likely to be primarily used for olfaction, and the other five for contact chemoreception (Schoonhoven and Van Loon 2002).

Gustation is a highly important sense for lepidopteran larvae, providing them with a rich quantity and quality of information (Glendinning et al. 1998; Agnihotri et al. 2016). As the caterpillar bites into foliage, the released fluids wash over the maxillary palpi, contacting the gustatory receptors (Chapman 2003). Fructose and non-fructose sugar receptors aid in the identification of suitable, sugar-rich food plants, triggering an enhanced feeding response; whereas receptors for bitter compounds protect larvae against potentially toxic plant secondary metabolites, their activation triggering a rapid cessation of feeding (Chapman 2003; Agnihotri et al. 2016). As such, neurons associated with gustatory receptors can broadly be referred to as either phagostimulatory or deterrent cells (Chapman 2003). Deterrent detection is more important to larvae than phagostimulant detection, likely because of the potential lethality posed by the former (Chapman 2003; Agnihotri et al. 2016). Even so, sensitivity to deterrents can vary widely between species, for example specialists are typically more refined in their responsiveness to deterrents than polyphagous generalist species (Bernays et al. 2000; Schoonhoven and Van Loon 2002; Sun et al. 2021). Conversely, specialist species’ deterrent cells do not respond to the secondary metabolites of their host plants, and this absence of deterrent reception is often the primary basis for host recognition (Schoonhoven and Van Loon 2002). As well as facilitating host recognition, these host-specific secondary compounds, which act as deterrents to polyphagous species, often act as phagostimulants for specialists (Van Loon and Schoonhoven 1999; del Campo et al. 2001; Schoonhoven and Van Loon 2002; Sun et al. 2021). Gustation of host-specific compounds during early instars also mediates induced host plant preference, subsequently making host-specific volatiles more attractive to larvae searching for food (Glendinning et al. 1998; Carlsson et al. 1999; del Campo et al. 2001).

Using olfaction, caterpillars are capable of perceiving and orienting towards volatiles from their preferred host plants (Carlsson et al. 1999; Carroll and Berenbaum 2002; Castrejon et al. 2006; Carroll et al. 2006; Glendinning et al. 2009; Rharrabe et al. 2014; de Fouchier et al. 2018), and selectively avoiding non-hosts based on olfactory cues (Piesik et al. 2009). This behaviour is likely facilitated by caterpillars’ ability to learn to associate odours with positive and negative stimuli (Salloum et al. 2011), although specialists (and less likely, generalists) also display an innate preference for host plant volatiles (Carlsson et al. 1999). A wide body of evidence suggests that, in many cases, it is specifically the volatiles of plants damaged by herbivory that larvae are preferentially attracted to (Landolt et al. 2000; Carroll et al. 2006, 2008; Mooney et al. 2009; Huang et al. 2009; McCormick et al. 2016), likely because these provide a reliable indicator of a suitable, non-toxic food source. In some cases, in a rather ironic twist, defensive compounds produced by the plant in response to herbivory damage, such as feeding deterrents and volatiles to attract natural enemies of larvae, are also the very olfactory attractants the larvae use to locate the plant (Carroll and Berenbaum 2002; Carroll et al. 2006; Huang et al. 2009; McCormick et al. 2016). Additionally, evidence suggests that larvae’s functional reception of odours, and their behavioural responses to certain odour cues, change as they age and gain experience feeding on their host, reflecting the different ecologies of the instars (McCormick et al. 2016; Revadi et al. 2021; Wang et al. 2024). For example, in some species, herbivory-induced plant volatiles are attractive to younger and/or naïve larvae but not to older and/or experienced larvae (McCormick et al. 2016; Revadi et al. 2021). This is likely because early instars are at the greatest risk of starvation and must quickly locate a suitable feeding site (Revadi et al. 2021), whereas older and more experienced larvae are under less pressure to feed and may associate herbivory-induced volatiles with increased competition and heightened plant toxin defences (McCormick et al. 2016; de Fouchier et al. 2018). *Helicoverpa armigera* (Noctuidae) caterpillars also respond to carbon dioxide (CO_2_) in the air, exhibiting behavioural attraction to higher CO_2_ concentrations, suggesting this gas may be utilised as a cue for finding food (Rasch and Rembold 1994). The sensors responsible for CO_2_ detection have been isolated to the maxillary palps (Keil 1996).

Aside from locating and gathering information on their host plants, caterpillar chemoreception is important for communication between conspecifics. The most well-known form of chemoreception-based communication between larvae is trail-following, whereby pheromone droplets, often deposited onto silk trails, are used to convey information about food patches (Fitzgerald and Edgerly 1979; Costa et al. 2001; Colasurdo and Despland 2005; Costa and Pierce 2010). This type of communication is well understood in the tent caterpillars of the Lasiocampidae family (Colasurdo and Despland 2005). These species construct large silk nests which they venture out of to forage. On their return from a foraging expedition, they will lay a pheromone trail from the patch to the nest for their nestmates to follow, much like the strategy used by many ant species. All species which use pheromone trails likely rely on contact chemoreception, not olfaction, to perceive the signals via their maxillary palps (Roessingh et al. 1988; Peterson and Fitzgerald 1991), yet the information carried in these pheromones, along with the caterpillar’s ability to perceive it, varies in complexity between species. Useful information that caterpillars can gather from such chemical signals include the age and strength of the pheromone, and whether it was laid by a conspecific and/or a nest mate that has recently fed (Roessingh et al. 1988; Roessingh 1989; Ruf et al. 2001; Fitzgerald 2003; Colasurdo and Despland 2005; Pescador-Rubio et al. 2011). Trail pheromones are also used by some processionary species for stragglers to find their way back to the procession (Fitzgerald and Pescador-Rubio 2002).

The use of trail pheromones for conspecific communication is widespread across larval Lepidoptera (Colasurdo and Despland 2005; Costa and Pierce 2010; Despland and Santacruz Endara 2016). However, whether caterpillars use any other forms of chemoreception-mediated communication is currently unknown. As well as host-specific volatiles, *Spodoptera littoralis* (Noctuidae) caterpillars have been found to show an attraction response towards conspecific female sex pheromones, which may be an adaptation to help them locate suitable hosts for feeding (Poivet et al. 2012). This behavioural response to olfactory pheromone cues released by a conspecific, along with the ability of many species to discern relatively complex information from conspecific pheromone signals, raises the question of whether caterpillars use olfactory pheromone signalling in other ecological contexts, such as alarm signalling. Larvae perform defensive behaviours when threatened which are effective at preventing attack from parasitoids (Gentry and Dyer 2002; Greeney et al. 2012). Thus, an alarm signal which induces these defensive behaviours could be selected for, especially in the case of gregarious species which stand to gain primary and secondary benefits by improving the survival of their sibling group members. However, this type of alarm signalling does not necessarily require the use of pheromones, and odorant release may even be selected against given that it could be exploited by natural enemies of the caterpillars to locate them (Saavedra and Amo 2018).

The lack of evidence for caterpillar odorant signalling, along with the potential for predator and parasitoid ‘eavesdropping’ to evolve, leads to the conclusion that it is unlikely that this type of communication between larvae is selected for in most contexts. However, it may be that caterpillars can perceive stress-induced olfactory cues from conspecifics as a warning. When threatened, some gregarious species regurgitate a noxious fluid that is repellent to attacking ants (Peterson et al. 1987; Müller et al. 2003). It is plausible that the odour of this fluid is detectable by conspecifics, and may or may not elicit a behavioural response. Indeed, in other insect lineages, defensive chemicals which are excreted in response to an attack have evolved from passively detected cues into sophisticated alarm signals (Leonhardt et al. 2016). Whether detection of group members’ regurgitate triggers larvae to perform this costly behaviour themselves (Higginson et al. 2011) is currently unknown, but offers a relatively straightforward avenue for future study.

### Thermoreception (thermal sensing)

Caterpillars are seemingly also capable of thermoreception, the sensation of temperature cues. Rapid behavioural responses, for example fleeing or moving body parts, have been observed in response to both radiative and conductive thermal stimuli (Frings 1945), however, as with sensitivity to touch, some of these observations are likely a nociceptive reaction (Frings 1945; Caron et al. 2020; Mukherjee and Trimmer 2020), rather than a truly thermoreceptive one. Nonetheless, some of the reactions observed were in response to warm and cold stimuli that may not have been extreme enough to trigger nociception (Frings 1945), and thus could be indicative of true thermoreception.

Many caterpillars are also known to exhibit thermoregulatory and perhaps thermotactic behaviours, such as seeking thermal refuges or reorienting their bodies relative to solar radiation in response to excessive or insufficient body temperatures (Casey 1976; Rawlins and Lederhouse 1981; Kevan et al. 1982; Karban 1998; Ruf and Fiedler 2002a; Bennett et al. 2003; Kührt et al. 2005; Nice and Fordyce 2006; Nielsen and Papaj 2015, 2017; Nielsen 2017; Nielsen et al. 2018; Uemura et al. 2021b). It is likely that these behaviours are at least sometimes mediated by some kind of thermoreceptor. However, the refuge-seeking behaviour of *Battus philenor* (Papilionidae) is triggered by surpassing a threshold body temperature, irrespective of whether the cue is radiative or conductive heat (Nielsen et al. 2018), suggesting any thermoreceptor involved in triggering refuge seeking is internal rather than external. In contrast, the process of finding the refuges, as well as the solar reorientation behaviours described, may be explained by an external thermoreceptor, but also other sensory modalities.

Mechanistically, thermoreception in caterpillars could be mediated by transient receptor potential (TRP) channels, which are implicated in thermoreception and thermotaxis by other larval and adult insects (Viswanath et al. 2003; Rosenzweig et al. 2005, 2008; Hamada et al. 2008; Wang et al. 2009; Kwon et al. 2010; Kohno et al. 2010; Gallio et al. 2011; Mao et al. 2020; Omelchenko et al. 2022), and are encoded for in the lepidopteran genome (Matsuura et al. 2009; Sato et al. 2014; Mao et al. 2020). As mentioned within the section on touch, nociceptive neurons respond to both noxious thermal and noxious mechanical stimuli in *Manduca sexta* (Sphingidae) caterpillars (Caron et al. 2020), although again the connections between detection of noxious stimuli and non-noxious stimuli in caterpillars are not yet clear.

### Hygroreception (humidity sensing)

One sensory modality that is often neglected in scientific research on caterpillars and animals more broadly, is hygroreception. Hygroreception refers to the detection of environmental humidity and is thought to be widespread amongst terrestrial arthropods (Rowley and Hanson 2007; Merrick and Filingeri 2019). Hygroreceptors have been identified in multiple adult insect species as tripartite sensilla, consisting of a ‘moist”’ sensory cell that responds to increases in relative humidity, a ‘dry’ sensory cell that responds to decreases in relative humidity, and a thermoreceptor (Steinbrecht and Müller 1991; Rowley and Hanson 2007; Merrick and Filingeri 2019). The exact mechanism of humidity detection by these sensory structures has not been conclusively identified, however, the most popular model suggests that hygroreceptors are modified mechanoreceptors that detect humidity via the mechanical forces induced during swelling and shrinking of hygroscopic material within the sensilla as water is absorbed or lost to the environment (Yokohari 1978; Steinbrecht and Müller 1991; Tichy and Gingl 2001; Rowley and Hanson 2007; Tichy and Kallina 2010; Merrick and Filingeri 2019).

As previously stated, studies on caterpillar hygroreception are limited in number, but in the larvae of *Manduca sexta* (Sphingidae), *Pieris rapae* (Pieridae), and *Heliothis xea* (Noctuidae), hygroreceptors have been electrophysiologically and behaviourally isolated to the antennae (Dethier and Schoonhoven 1968; Rowley and Hanson 2007). Others have been putatively identified on anatomical grounds in *Homoeosoma nebulella* (Pyralidae) (Faucheux 1995) and *Dendrolimus kikuchii* (Lasiocampidae) (Men and Wu 2016). Interestingly, the hygroreceptors of caterpillars appear to only respond to moisture, and not to dryness, distinguishing them from the previously described system in adult insects (Rowley and Hanson 2007).

Given their vulnerability to desiccation and the impact of relative humidity on development (Jaco Klok and Chown 1997; Woods et al. 2000; Han et al. 2008), caterpillars likely use hygroreception to identify areas with sufficient moisture. Indeed, desiccated caterpillars placed in an arena with a humidity gradient preferred the areas of higher humidity, whereas normally hydrated caterpillars did not (Rowley and Hanson 2007). This preference was lost when the antennae were ablated or occluded (Rowley and Hanson 2007). Further, caterpillars with their antennae occluded also encountered a water droplet less frequently, and exhibited suppressed drinking behaviour when encountering water, suggesting that hygroreception may also mediate the search for and initiation of drinking water (Rowley and Hanson 2007). Lastly, it has been suggested that hygroreception could aid caterpillars in assessing the condition of leaves, as cut fresh leaves elicited an electrophysiological reaction that was not seen in response to cut wilted leaves (Dethier and Schoonhoven 1968).

### Graviception (gravity sensing)

A ubiquitous force on Earth is gravity, and thus it is unsurprising that the detection of gravity is a sense seemingly found throughout much of the animal kingdom. Naturally, this extends to caterpillars, which stand to benefit from using gravity as a reliable and omnipresent sensory cue for guiding vertical locomotion and orientation behaviours. Indeed, geotactic preferences have been experimentally demonstrated in a number of caterpillar taxa (Bernays et al. 1985; Ramachandran 1988; Perkins et al. 2008). The identities of the putative gravity sensors in caterpillars have not been extensively interrogated (van Griethuijsen and Trimmer 2009), but numerous specialised and unspecialised mechanosensory-based graviceptors already identified in other insects are strong candidates (Bender and Frye 2009).

## The threat of sensory pollution

With the plethora of senses that caterpillars are known to be endowed (Fig. 1), it is an unfortunate inevitability in the modern day that caterpillars will be negatively impacted by anthropogenic sensory pollution in some or all of these modalities.

### Light pollution

When most people think of sensory pollutants, often the first that comes is artificial light at night (ALAN). ALAN is a sensory pollutant with the potential to affect both diurnal and nocturnal caterpillars and appears to be a driver of global Lepidoptera declines (Boyes et al. 2021a, b). The impacts on the behaviour and physiology of caterpillars are not uniform and change in the directionality of effect depending on the characteristics of the light (van Geffen et al. 2014), species (Van de Schoot et al. 2025), sex (van Geffen et al. 2014; Van de Schoot et al. 2025), and geographic location (Péter et al. 2020; Merckx et al. 2023). Generally, the mechanism driving the effects of light pollution on caterpillars is related to the disruption of the circadian and seasonal synchronisation of fundamental biological processes with the cyclical change in ambient light from day to night. Artificial light pollution alters the photoperiod (day length) of the 24-hour day/night cycle (Gaston et al. 2017) and increases the brightness of the night sky (Kyba et al. 2017). This can extend the perceived day length and mask the natural changes in ambient light levels that are responsible for triggering or suppressing important physiological and behavioural processes. In caterpillars, feeding and development (Ruf and Fiedler 2002b), diapause (Peterson and Hamner 1968; Hayes et al. 1970; Hasegawa and Shimizu 1987; Huang et al. 2005; He et al. 2009; Chen et al. 2011; Yang et al. 2014; Ahmadi et al. 2018), pupation (van Geffen et al. 2014), and the expression of some hormones (Kim et al. 2019) are all entrained to the 24-hour light cycle, as well as the reciprocal changes in temperature in some instances (Peterson and Hamner 1968). The disruption of diel and seasonal light can cause shifts in the feeding (Haynes et al. 2023; Van de Schoot et al. 2025) and activity (Schroer et al. 2019) patterns of caterpillars and effect developmental traits such as body mass (van Geffen et al. 2014; Grenis and Murphy 2019; Van de Schoot et al. 2025), timing and duration of diapause (Hayes et al. 1970; Schroer et al. 2019; Merckx et al. 2023) and pupation (van Geffen et al. 2014; Schroer et al. 2019; Van de Schoot et al. 2025). This could lead to reduced fecundity in females (Van de Schoot et al. 2025), greater annual generations and local infestations, and asynchronisation with the meteorological seasons causing local extinctions. Although, more generalist species (Van de Schoot et al. 2025) or species with populations in extreme latitudes (Merckx et al. 2023), and thus preadapted to extended photoperiods, may have a higher potential for adaptation to light pollution.

Observations from field-based experiments show that light pollution can affect the spatial and temporal activity of invertebrate predators and thus caterpillar survival, though again, with mixed directionality in effects. In mixed grass prairie and grassland mesocosms, the presence of streetlights or simulated skyglow had no effect on predation on wax worm (*Galleria mellonella*, Pyralidae) caterpillars (Grenis et al. 2015) or clay caterpillar models (Dyer et al. 2023). However, the latter study did observe an increase in the nocturnal activity of predators which could lead to increased predator-prey encounters in lit grasslands. In light-naïve forests, the presence of artificial light at night of 10–15 lx (similar to a typical residential streetlight in the United Kingdom (British Standards Institution 2015) caused an increase in predator abundance and predation rates on model caterpillars (Deitsch and Kaiser 2023). The authors attribute some of these discrepancies to differences in the spectral composition and intensity of the lighting technologies used, the species composition of arthropod communities under observation, and local differences in the levels of background light pollution. Importantly though, these effects are not a result of direct sensory pollution on the caterpillar’s visual sense, but nonetheless may introduce new and more intense challenges to the anti-predator sensory modalities of caterpillars, primarily hearing, vibration detection, electroreception, and touch. While much remains unknown about the ecological and evolutionary effects of light pollution on caterpillars, especially direct effects on their visual system, the potential impacts could be substantial.

### Noise pollution

In the modality of hearing, there is also evidence of the negative impact of anthropogenic sensory pollution. Monarch butterfly caterpillars (*Danaus plexippus*, Nymphalidae) exhibit raised heart rates in response to playbacks of noise from highways, suggestive of increased stress resulting from detection of the sounds (Davis et al. 2018). This is likely because the frequency spectrum of the road noise overlaps with that of the wingbeat of caterpillar predators, thus noise pollution may also lead to habituation or desensitisation of caterpillars to the real acoustic cues of their predators. Furthermore, the same species has been noted to react defensively to the sound of passing jet aircraft (Rothschild and Bergström 1997). Beyond this, surprisingly few studies address noise pollution as a threat to caterpillars, though it has been noted that exposure to the flight sounds of predators and bees causes caterpillars to eat less and accelerate their development (Tautz and Rostás 2008; Lee et al. 2021), which hinders their survival. It is likely that anthropogenic sound sources, such as road noise, would also trigger these effects, and thus the ways in which anthropogenic noise threatens caterpillars are manifold.

Vibration detection remains unexplored in relation to anthropogenic sensory pollution, but it is highly conceivable that the same sources of anthropogenic noise in airborne sound could also elicit spurious vibrations in the substrate. Thus, human activity may have similar detrimental effects on caterpillars in the vibratory sense as occurs through hearing.

### Electrostatic pollution

Another emerging threat is within the sense of aerial electroreception. As a relatively newly discovered sense in any animal (Clarke et al. 2013; England and Robert 2022, 2024a), the possibility that anthropogenic sources of electricity, or human perturbations of natural electric fields, could have an adverse ecological impact remains to be explored. Evidence in bees shows that the application agricultural chemicals to flowers modifies their electric field and reduces visitation by bumblebees (Hunting et al. 2022a). As regular inhabitants of agricultural and horticultural landscapes, the possibility that electroreception in caterpillars could also be hindered by the application of such chemicals should be considered. Furthermore, the electromechanical resonance of caterpillar setae includes 50 and 60 Hz (England and Robert 2024a), which are the frequencies of mains electricity and most powerlines in the world. As such, caterpillars situated near to anthropogenic power infrastructure, including powerlines and urban areas, are likely also detecting these anthropogenic electric fields. This has the potential to greatly hinder caterpillars’ ability to detect their predators via electroreception, either through habituation or saturation, especially given that the magnitude of anthropogenic electric fields is often orders of magnitude higher than natural sources (England and Robert 2022). Indeed, evidence in bees suggests that the electrostatic fields of powerlines reduce floral landings, perhaps due to sensory pollution in their electroreceptive sense (Mallinson et al. 2025). Furthermore, it has been shown that high voltage powerlines modify the electrical environment, via the introduction of excess charges, for over 1 km in their vicinity (Matthews et al. 2024). This may modify the charges accumulated by organisms and thus potentially compromise the reliability of the caterpillar electroreceptive system. Future work should aim to directly address these hypotheses and assess the extent to which anthropogenic electricity is a sensory pollutant for caterpillars and other organisms.

### Chemosensory pollution

Chemoreception is another sensory modality in which anthropogenic pollution likely hinders survival of caterpillars, but very little has been studied explicitly in this regard to date. Given that humans release great quantities of foreign chemicals into the environment, it seems highly likely that these would at times be detected chemically by caterpillars and trigger maladaptive behavioural responses. Additionally, exposure to certain chemicals may desensitise or modify the chemical sense in caterpillars. Indeed, *Helicoverpa armigera* (Noctuidae) caterpillars raised on diets containing deterrent chemicals became less sensitive to these chemicals at both the behavioural level and neuronal level in the gustation receptors of the maxillae (Zhou et al. 2010). Furthermore, it has been shown outside the Lepidoptera, in adult houseflies (*Musca domestica*), that contamination of the antennae with anthropogenic particulate matter compromises the perception of olfactory cues (Wang et al. 2023). It is very plausible that such effects also apply to olfaction by caterpillars.

### Heat and humidity pollution

Nothing is currently known about whether anthropogenic heat and humidity sources, such as those associated with buildings and infrastructure, could interfere with caterpillars’ senses of thermoreception and hygroreception. Since both humidity and heat cues can trigger relocation behaviours, it is conceivable that anthropogenic sources could confuse caterpillars and cause them to move to less optimal feeding or pupation sites, misled by our spurious modification of the local climatic conditions. These ideas deserve empirical investigation to assess the possibility of human activity hurting lepidopteran survival in this way.

## Conclusions and the value of caterpillars to neuroethology

Overall, it is clear that caterpillars inhabit a rich sensory Umwelt, on which great progress has been made in our collective understanding of their ecology and neuroethology. Despite this, there persist many notable knowledge gaps and open questions which certainly warrant exploration in the future. Aiming to answer these questions promises to not only enhance knowledge of caterpillar biology, but also principles of sensory ecology and evolution more broadly.

Firstly, the mechanisms of multiple sensory modalities await conclusive identification in caterpillars. In particular, the mechanism of vibration detection should be tested by electrophysiological means, especially given the existence of many strong candidate receptors (Yack 2022; Yack and Yadav 2022). Similarly, whilst the electromechanical responses of mechanosensory setae to electrostatic fields strongly point to their role in electroreception in caterpillars (England and Robert 2024a), this should also be formally confirmed with electrophysiology. The same is true for identifying if and how caterpillars truly sense touch directly on the body wall (Frings 1945). Likewise, future work should aim to revisit earlier experiments on thermoreception to confirm sensitivity to non-noxious thermal stimuli (Frings 1945), and if validated, identify the receptors involved. Lastly, although magnetoreception remains to be observed in caterpillars, the possibility deserves investigation, with long-distance navigators like processionary caterpillars being key candidates. The mechanisms of magnetoreception have not yet been conclusively identified in any animal (Johnsen and Lohmann 2005; Nordmann et al. 2017), and so given the unique attributes that caterpillars bring to neuroethological research, any discovery of magnetoreception in caterpillars could open the door for them to contribute to solving this ongoing enigma.

Another matter of urgency that emerges from this review is the lack of specific and mechanistic data on how anthropogenic sensory pollution may affect caterpillars across all sensory modalities, especially the newly discovered sense of electroreception. Whilst some information is known on how light, sound, and chemical pollution may interfere with their respective sensory modalities in caterpillars, much more needs to be done, especially to establish less inferential evidence and identify more direct and proximate impacts on the caterpillars themselves. For example, despite the great amount of data demonstrating phototaxis in caterpillars, there is a dearth of studies explicitly exploring how this behaviour may be confused by anthropogenic light sources. In the many other sensory modalities of caterpillars, such as vibration detection, electroreception, hygroreception, and potentially thermoreception, essentially nothing is known of the impacts of sensory pollution. Whilst predictions can be made in this regard, effective conservation and management of the world’s Lepidoptera will necessitate rapid formal scientific identification of any such effects, so that suitable mitigation strategies can be formulated and tested. It is important that we do not neglect the caterpillar stage of their lifestyle in these efforts.

Many valuable aspects of caterpillar biology are yet to be utilised to the advantage of sensory ecology and neuroethology research. Firstly, despite the recent convergence on a somewhat resolved phylogenetic tree for the entire order (Bazinet et al. 2013, 2017; Kawahara and Breinholt 2014; Kawahara et al. 2019; Mayer et al. 2021) (though see (Rota et al. 2022)), in addition to many phylogenies for finer taxonomic resolution in specific groups, there are essentially zero studies to date that have leveraged these to take a comparative approach to the study of caterpillar sensory systems. Such a comparative approach has proven fruitful in the study of adult lepidopteran sensory systems (Kawahara et al. 2019; Wainwright et al. 2025), and in non-sensory traits of caterpillars (Hossie et al. 2015; McLellan et al. 2023a), and so will surely reveal new and exciting principles of sensory system evolution and its ecological correlates in caterpillars.

Furthermore, it has been suggested in a few lepidopteran families that memories and sensory experiences acquired during the caterpillar stage survive metamorphosis and can influence adult behaviour (Akhtar and Isman 2003; Chow et al. 2005; Olsson et al. 2006; Blackiston et al. 2008; Shikano and Isman 2009; Sant’Ana and Gregório 2016; Sant’Ana et al. 2021). This has been most convincingly demonstrated in the retention of memory through metamorphosis of olfactory associative learning at the caterpillar stages of *Manduca sexta* (Sphingidae) and *Grapholita molesta* (Torticidae) (Blackiston et al. 2008; Sant’Ana et al. 2021). This ability, especially if widespread within the order, presents a unique dimension with which to test neuroethological questions in both basic and applied (conservation and management) science, and should be capitalised on in future work.

Lastly, it is noteworthy that caterpillars present an excellent example of sensory versatility in the function of their mechanosensory setae. So far, it has been identified that the mechanosensory setae of caterpillars are implicated in the sensation of airborne sound (hearing), electric fields (electroreception), contact (touch), and potentially substrate-borne sound (vibration detection). Whilst each seta is likely, to some extent, multimodal in this regard, it is also probable that specific setae are specialised for each sensory modality. Therefore, the evolution of these multiple sensory capacities from a single, relatively simple, sensor type offers a fantastic opportunity to explore how sensilla geometry, biomechanics, and material properties can enhance sensitivity to certain stimuli, and perhaps reduce noise from others. Thus, in this way, caterpillars present a strong model system for investigations into sensory specialisation, sensor design, and multimodal integration.

Altogether, it is evident that a strong mutual benefit exists between the study of caterpillars and the study of sensory ecology. It is hoped that this review raises awareness of and strengthens this mutual benefit further, facilitating future research into this area. Such work stands to reveal and progress scientific understanding, not only of pure ecology and evolution, but also applied conservation and management, ideally working towards a stable future for the Lepidoptera and the ecosystems within which they play a vital role.

## Data Availability

No datasets were generated or analysed during the current study.
